# Oropharyngeal administration of mother’s own milk influences levels of salivary sIgA in preterm infants fed by gastric tube

**DOI:** 10.1038/s41598-022-06243-2

**Published:** 2022-02-09

**Authors:** Li-Lian Chen, Jie Liu, Xiao-He Mu, Xi-Yang Zhang, Chuan-Zhong Yang, Xiao-Yun Xiong, Mei-Qi Wang

**Affiliations:** 1 Department of Neonatology, Affiliated Shenzhen Maternity & Child Healthcare Hospital, Southern Medical University, Shenzhen, China; 2 Shanxi University of Chinese Medicine, Shanxi, China

**Keywords:** Paediatric research, Randomized controlled trials, Immunology

## Abstract

The aim of the present study was to explore the effect of oropharyngeal mother’s milk administration on salivary secretory immunoglobulin A (sIgA) levels in preterm infants fed by gastric tube. Infants (n = 130) with birth weight < 1500 g were randomly allocated into two groups which both received breast milk for enteral nutrition. The experimental group (n = 65) accepted oropharyngeal mother’s milk administration before gastric tube feeding for 14 days after birth. The control group (n = 65) accepted oropharyngeal 0.9% normal saline administration. Saliva concentration of sIgA were assessed at the 2 h, 7th and 14th day after birth. The level of salivary sIgA in experimental group were significantly higher than those in control group on the 7th day after birth (p < 0.05), but there were no differences in salivary sIgA levels on the 14th day between the two groups. The results of quantile regression analysis showed that oropharyngeal mother’s milk administration, delivery mode and gestational age had significant effects on the increase of sIgA. SIgA in experimental group and the total number of intervention had a significant positive correlation (p < 0.05). Oropharyngeal mother’s milk administration can improve salivary sIgA levels of preterm infants.

## Introduction

Premature birth complications are now the second leading reason for death in children under 5 years of age^1,2^. Small for gestational age, especially severe small for gestational age, is ralated to an increased risk of death, with infectious diseases being one of the largest causes of death^3,4^. Action is required to increase survival and reduce mortality in preterm, exceptional immaturity of infants.

Breast milk is the first immune stimulant for infants, which contains very rich immune substances, such as lactoferrin, immunoglobulin, oligosaccharide^5^. Secretory immunoglobulin A (sIgA) is one of the most important antibody for human mucosal immunity^6–8^. In addition to maintaining a symbiotic relationship with the indigenous microbiota, sIgA directly prevents pathogens from adhering to epithelial cells, thus achieving a local anti-infection effect^9,10^. During the common process of oral feeding, the oral mucosa contacts with sIgA in mother’s milk, which helps to establish of mucosal immune barrier against pathogens^11^. Uncoordinated sucking and swallowing in preterm infants makes oral breastfeeding difficult, especially those with low birth weight and small gestational weeks, they still need gastric tube feeding for several days or weeks after birth^12^. The oral mucosa of preterm infants fed by gastric tube can not get access to breast milk, there will be a lot of pathogenic bacteria in the oral cavity, which is easy to cause local or systemic infection^13^. Several studies indicated that nurses could prepare syringe for breast milk instillation or sterile swabs with human milk to wipe the mouth of premature infants^14,15^. This intervention is also known as Oral Immune Therapy. Another study reported that administration of oropharyngeal mother’s milk to preterm infants (< 32 weeks gestation) had a significantly lower growth of Klebsiella species in the oropharyngeal^16^. There are several hypotheses suggested that oropharyngeal mother’s milk administration works as an immunomodulatory agent through several mechanisms: (1) the immune factors in breast milk interact with the immune cells of the oropharyngeal-associated lymphoid tissue and bronchial-associated lymphoid tissues^17^. (2) The oral mucosa absorbs protective biofactors, and the blood flow-rich capillaries in the mucosa promote the absorption of biofactors into the systemic circulation. (3) The contact between oropharynx and breast milk facilitates the formation of a local mucosal protective barrier to prevent the colonization of pathogenic bacteria^18,19^. However, these hypotheses are not supported by evidence, and the mechanism of oropharyngeal breast milk administration needs to be further investigated.

The purpose of this study was to investigate the effect of oropharyngeal mother’s milk administration on salivary sIgA in preterm infants fed by gastric tube, and to provide a theoretical basis for clinical application.

## Results

**Figure 1:**
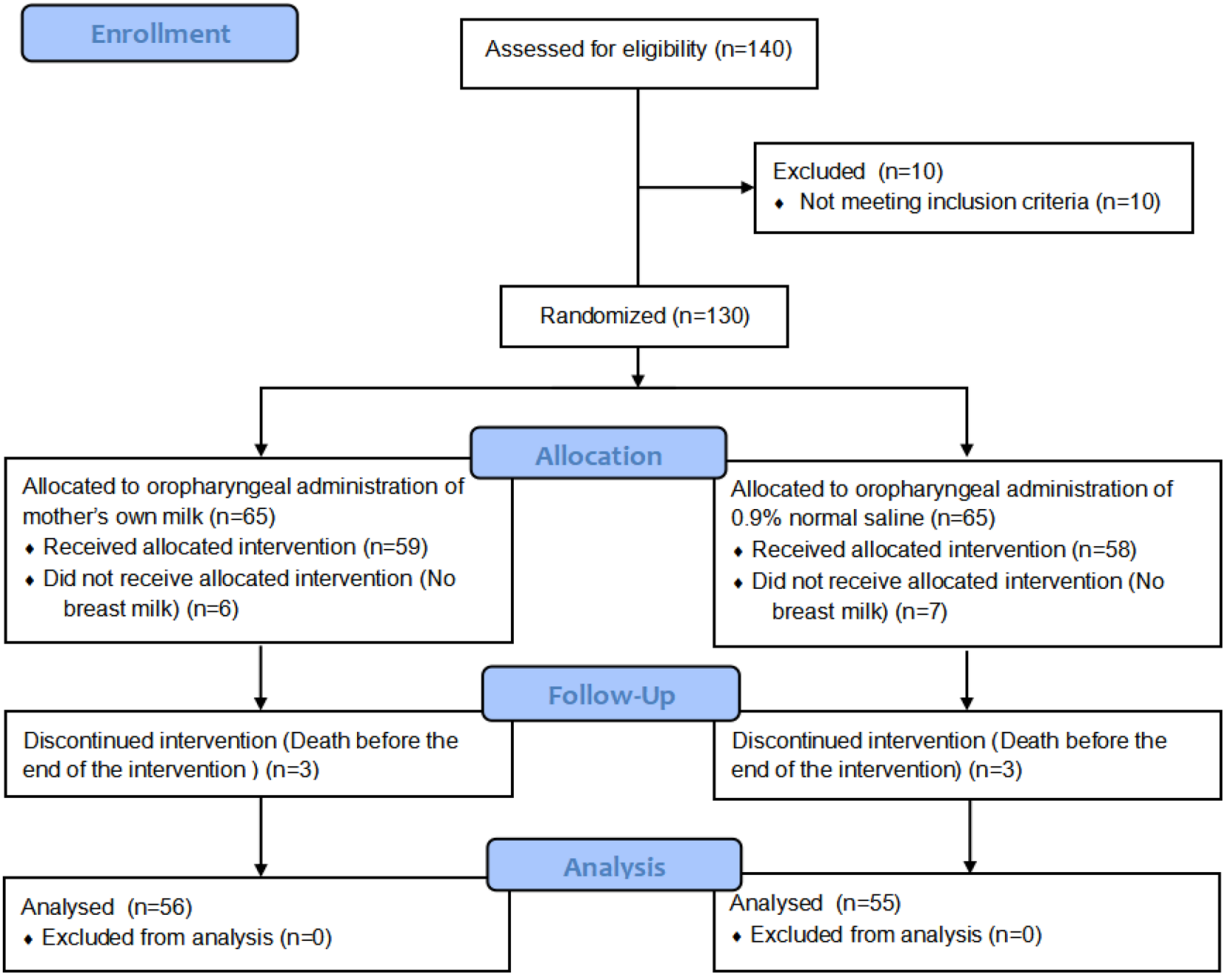
Flow diagram of the study population.

A total of 130 subjects were enrolled in the study. Among the participants, 9 infants in the experimental group and 10 infants in the control group dropped out because they had no milk availability after 96 h postpartum or died before the end of the intervention (Fig. 1). No significant (*p* > 0.05) differences were found in the baseline characteristics between both groups (Table 1).

**Table 1:** Population characteristics.

Infant characteristics	Study group	Control group	*t/χ*^2^	*p *value
**Gender n (%)**			0.451	0.502
Male	28 (50)	31 (56)		
Female	28 (50)	24 (44)		
Gestational age at birth (eewk, M ± SE)^1^	29.48 ± 1.83	29.84 ± 2.19	− 0.930	0.355
Birth weight (kg, M ± SE)	1.24 ± 0.25	1.17 ± 0.21	1.611	0.110
Apgar score at 1 min (M ± SE)	8.50 ± 2.20	8.49 ± 2.28	0.021	0.983
Apgar score at 5 min (M ± SE)	9.69 ± 1.43	9.67 ± 1.32	0.090	0.928
**Mode of delivery n (%)**			0.015	0.901
Vaginal delivery	21 (37.5)	20 (36.4)		
Cesarean delivery	35 (62.5)	35 (63.6)		
**Twins** **n (%)**			2.022	0.155
Yes	18 (32.1)	27 (49.1)		
No	38 (67.9)	28 (50.9)		
Invasive mechanical ventilation	9 (16.1)	10 (18.2)	0.087	0.768
Time of start enteral feeding with MOM^2^ (h, M ± SE)	58.04 ± 33.97	59.78 ± 34.92	− 0.26007	0.790
**Feeding for 2wk after birth**			0.073	0.788
Breast milk	20 (35.7)	21 (38.2)		
Mixed	36 (64.3)	34 (60.7)		
**Maternal characteristics**				
Age (year, M ± SE)	30.51 ± 4.56	32.70 ± 8.31	− 1.720	0.087
Gestational diabetes n (%)	12 (21.4)	14 (25.5)	0.251	0.617
Gestational hypertension n (%)	6 (10.7)	12 (21.8)	2.518	0.113
Placental abruption n (%)	5 (8.9)	3 (5.5)	0.501	0.479
Premature rupture of membranes n (%)	29 (51.8)	21 (38.2)	2.074	0.150
Antenatal steroid n (%)	47 (83.9)	49 (86.5)	0.633	0.426
Antenatal magnesium sulfate n (%)	15 (26.8)	12 (24.3)	0.372	0.542

Values were expressed as the means (M) ± standard error (SE) or number (%). ^2^MOM, mother’s own milk; t, Independent samples t-test; χ^2^, Chi-square test.

There were no adverse effects during the protocol treatment. Breastfeeding initiation times were (58.04 ± 33.97) and (59.78 ± 34.92) hours after birth in the experimental and control groups, respectively, with no statistically significant differences. The start of oropharyngeal administration in the experimental group was consistent with the beginning of breastfeeding. The number of interventions completed within 14 days for infants in the experimental group was 80.38 ± 15.29.

Table 2 presented the concentration of salivary sIgA in experimental group were significantly higher than those in control group on the 7th day after birth. The difference was statistically significant (p < 0.05), but there were no differences in salivary sIgA levels on the 14th day after birth between the experimental and control group.

**Table 2:** The concentration of salivary sIgA of the study population (μg/ml).

	Study group	Control group	*p* value
Day1	0.487 (0–11.075)	0 (0–4.677)	0.076
Day7	29.809 (0.687–140.257)	0 (0–3.219)	0.000
Day14	45.616 (6.771–146.028)	13.368 (0.740–72.734)	0.140

Datas were expressed as M (P25 ~ P75).

**Figure 2:**
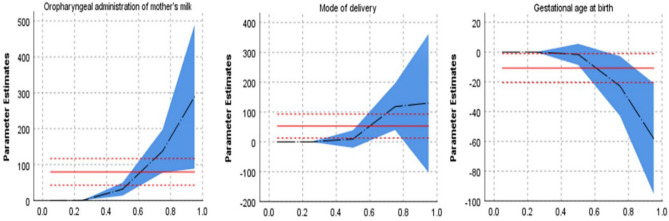
Association of sIgA with oropharyngeal administration of mother’s milk, mode of delivery, gestational age at birth. The graphs sequentially represent the 95% confidence intervals and beta values of the regression coefficients for oropharyngeal administration of mother’s milk, mode of delivery, gestational age at birth.

Table 3 showed the quantile regression analysis of the influencing factors of salivary sIgA in the two groups on the 7th day after birth. Infant gender, birth weight, twins, Apgar score, time of starting enteral feeding with MOM, maternal gestational diabetes, gestational hypertension, antenatal steroid and antenatal magnesium sulfate had no effect on sIgA levels in saliva. The regression coefficients of oropharyngeal administration of mother’s milk on P25, P50, P75 and P95 were statistically significant (p < 0.05). The regression coefficients of mode of delivery on P75 were statistically significant (p < 0.05). The regression coefficients of gestational age at birth on P75 and P95 were statistically significant (p < 0.05). The 95% confidence intervals of regression coefficients were shown in Fig. 2.

**Table 3:** The quantile regression analysis of the influencing factors of salivary sIgA in the two groups on the 7th day after birth.

	*P*_5_		*P*_25_		*P*_50_		*P*_75_		*P*_95_	
	*β*	*p*	*β*	*p*	*β*	*p*	*β*	*p*	*β*	*p*
Intervention	0	……	0.690	0	31.770	0.001	137.660	0	289.160	0.005
Mode of delivery	0	……	0	1	9.810	0.510	118.240	0.003	129.930	0.270
Gestational age at birth	0	……	0	1	− 1.497	0.676	− 22.882	0.026	− 58.047	0.002

*P5* 5th percentile of sIgA concentration, *P25* 25th percentile of sIgA concentration, *P50* 50th percentile of sIgA concentration, *P75* 75th percentile of sIgA concentration, *P95* 95th percentile of sIgA concentration, *β* beta value.

In experimental group, Salivary sIgA level and the total number of oropharyngeal administration of mother’s milk had significant positive correlation (r = 0.280, p = 0.037) within 7 days of birth. There was also a significant positive correlation between the two for the first 14 days of life (r = 0.331, p = 0.013).

Table 4 showed that there was no significant difference in the clinical results of Necrotizing enterocolitis (NEC), Ventialtor-associated pneumonia (VAP) and Late-onset sepsis (LOS) between the two groups. The time of full oral feeding in experimental group were significantly shorter than those in control group.

**Table 4:** Clinical outcomes between two groups at the time of discharge.

	Study group	Control group	*t/χ*^2^	*p*
Necrotizing enterocolitis	5 (8.92)	3 (5.45)	0.501	0.479
Ventialtor-associated pneumonia	3 (5.36)	4 (7.27)	0.172	0.678
Late-onset sepsis	3 (5.36)	1 (1.82)	1.000	0.317
Intraventricular hemorrhage	8 (14.3)	13 (23.6)	1.582	0.209
Alimentary tract hemorrhage	1 (1.79)	5 (9.01)	2.896	0.089
Feeding intolerance	4 (7.14)	10 (18.2)	3.068	0.080
Time of full oral feeding	35.385 ± 1.191	36.030 ± 1.712	− 2.220	0.029
Hospital stay	54.154 ± 17.923	58.647 ± 24.091	− 1.075	0.285

## Discussion

This study was to evaluate the effect of oropharyngeal mother’s milk administration on salivary sIgA levels in infants fed by gastric tube. This intervention is available and safe for very low birth weight (VLBW) infants. We found that the difference in salivary sIgA concentrations between the two groups on the 7th postnatal day was significant, but the difference on the 14th day was not significant. Of note, salivary sIgA was positively associated with the total number of oropharyngeal mother’s milk administration. We also found that sIgA in saliva was associated with the mode of delivery and gestational age at birth in addition to oropharyngeal mother’s milk administration.

SIgA is a dimer that can transported through the mucosal epithelium and constitutes the first immune barrier against the invasion of harmful substances. In addition to protecting against mucosal infections, sIgA also plays a role in regulating phagocytosis and neutralizing toxins^20–22^. Administration of oropharyngeal milk is an effective way to expose the oral mucosa of preterm infants to the immunoglobulins in breast milk^23^. In our study, the level of salivary sIgA in experimental group were higher than those in control group on the 7th day after birth, the difference was statistically significant (p < 0.05) (Table 2). This result could explain the conclusion of similar trials that sIgA is increased in the saliva of VLBW infants in the presence of the administration of oropharyngeal colostrum^24^. Quantile regression analysis showed that the beta value increased from 0.69 to 289.16 with increasing sIgA levels percentile. It is plausible that the sIgA in breast milk is not only absorbed, but also stimulates the immune system leading to active immunity when the oral mucosa is exposed to breast milk^25^. Moreover, the immune components in human milk may interact with the immune cells in the oropharyngeal-associated lymphoid tissue and bronchial-associated lymphoid tissues to promote the maturation of the local immune system^26^.

When comparing experimental and control group, there were no differences in salivary sIgA on the 14th day after birth. In comparison with the study by lee et al.^27^, the results were similar, although the intervention in our study was continued until 14 days after the birth of infants. Oropharyngeal administration of transition milk did not have a significant effect on the salivary sIgA. There was increasing trend in sIgA on the 14th day after birth in both groups, this find may have been related to the growth and development of preterm infants. The correlation analysis in this study showed that the total number of interventions was positively correlated with the levels of sIgA in saliva in the study group, suggesting that the greater number of oropharyngeal mother’s milk, the greater sIgA in saliva. At present, there is no consistent operational procedure for oropharyngeal administration of mother’s milk. The frequency of intervention varies from 2 to 6 h, and the duration varies from 48 h to 15 days or until oral feeding. It is necessary to further emphasize the importance of standardized process for oropharyngeal administration.

The mode of delivery was closely related to the development of premature infant. The results of quantile regression analysis in this study showed that the mode of delivery was one of the factors that affect the sIgA, especially at the P75 percentile (p < 0.05). Two reasons might explain these differences. First, lactobacillus, Prevotella and Gardnerella were the most abundant genera in the vaginal delivery infant oral microflora. Pseudomonas, Staphylococcus were dominant bacteria in the cesarean delivery infant oral microflora^28^. Lactic Acid Bacteria can increase the levels of IgA secreting cells and IgA levels^29^. Second, compared with cesarean section, the start human milk secretion time of vaginal delivery women was earlier and milk production was more^30^. The concentration of immune substances in breast milk showed a decreasing trend with the extension of time. The incision pain after cesarean section led to the increase of sympathetic nerve excitation and the release of catecholamine, which inhibited the secretion of oxytocin and reduced milk secretion^31^. Cesarean delivery not only affects the oral flora of the infant, but also delays breast milk production and reduce sIgA in breast milk. SIgA in the saliva of preterm infants may be affected by all of these. Therefore, maternal vaginal delivery should be encouraged to facilitate early access to breast milk for newborns.

The results of quantile regression analysis in this study showed that the gestational age at birth is one of the factors that affect the sIgA, especially at the P75 and P95 percentiles (p < 0.05). β value changed from − 22.882 to − 58.047 with the increase of sIgA level percentile. This find may have been correlated with the sIgA levels in breast milk. Studies have shown that the duration of pregnancy is inversely related to the concentration of protective factors in breast milk^32,33^. This means that milk produced by mothers with extremely low birth weight infants has the highest concentration of the protective factor. Therefore, oropharyngeal administration of mother’s milk could be performed as early as possible for extremely premature infant.

The average time to start the breastfeeding in the experimental group was 58.04 ± 33.97 h after the infants’ birth, which was similar to the time of human milk obtained in the study of Zhang et al.^34^. The first reason for the difficulty in obtaining colostrum within 24 h after birth hospitalization of preterm infants may be related to the separation of mother and baby, which prevents early suckling to stimulate breast milk production. The second reason is that mothers of preterm infants often have perinatal complications and continue to need some medications that may affect breastfeeding after giving birth. These factors may affect the start time and frequency of oropharyngeal administration of mother’s milk. In order to make premature infants contact with breast milk as early as possible, many large human milk banks have been established in North America and some European countries, and relatively complete management guidelines for human milk banks have been established. At present, breast milk banks are established relatively late in China. Here, we suggest further improvement of the breast milk bank management system in order to shorten the time for infants to obtain colostrum. This may allow the implementation of oropharyngeal administration of mother’s milk for gastric tube feeding prematures as soon as possible.

The results of this study showed that oropharyngeal administration of mother’s milk had no effect on the incidence of NEC, VAP, Late-onset sepsis and length of hospital stay. A recent retrospective cohort study of oropharyngeal colostrum administration in premature infants every 4 h for 5 days did not find significant decrease in the incidence of NEC^35^. However, a systematic review and meta-analysis showed that oropharyngeal colostrum was associated with a significantly reduced incidence of VAP and a potential significance of NEC^36^. It is worth noting that although oropharyngeal administration of mother’s milk did not have a significant effect on clinical comorbidities, it did reduce the time of full oral feeding. Only four of the 56 infants in the experimental group suffered from feeding intolerance, while the control group had 10 infants. The results of Abd-Elgawad et al. were consistent with this study^16^. Protective biological factors in breast milk may provide local maturation at the mucosal surface, such as sIgA, lactoferrin and oligosaccharides prevent the adhesion and translocation of pathogenic bacteria into the intestinal mucosa, thus protecting against NEC. In addition, growth factors may also spread to the gastrointestinal tract, enhancing intestinal motility and reducing feed intolerance^37^.

Limitations of this study is that we cannot obtain breast milk within 24 h after birth for each baby and cannot control when the intervention starts due to the different lactation time of each mother. Therefore we recommend that breast milk banks should be established in each region to solve this problem. Further studies are necessary to investigate the frequency and start time of the oropharyngeal breast milk administration and its impact upon infants health.

## Conclusions

This study strengthens the notion that oropharyngeal administration of mother’s milk is safe and feasible intervention. The intervention can improve salivary sIgA levels of preterm infants, especially those delivered vaginally. We suggest extremely preterm infants to be frequent administrated of oropharyngeal breast milk.

## Materials and methods

### Ethics

The study protocol was approved by the Ethics Committee of the Shenzhen Maternity & Child Healthcare Hospital (SFYLS[2020]038). In addition, the clinical trial registration for this study is ChiCTR2100046645 (24/5/2021)in the Chinese Clinical Trial Registry. Parental informed consent was obtained within 2 h of birth. Parental informed consent was obtained before study enrollment for each infant.

### Subjects

Premature infants were eligible for the study who meet the following inclusion criteria: birth weight ≤ 1500 g and gestation ≤ 32 weeks, transferred to NICU within 2 h after birth, fed by gastric tube, parents agree to provide breast milk.

Exclusion criteria were: infants with congenital oral and gastrointestinal anomalies; maternal history of positive human immunodeficiency virus (HIV) status or syphilis; the mother being in the acute stage of infection that prevents her from providing breast milk, including severe mastitis, fungal infections of the breast or nipple that require treatment.

The shedding criteria were as follows: infant was requested to withdraw at any stage of the trial by parents; infant was transferred to other hospitals or died before the end of the trial; mothers can not provide colostrum for their infants in the first 96 h of life; breast milk tested positive cytomegalovirus.

### Collection of breast milk

Breast milk collection was completed by the maternal and her family members. On the day the infants were admitted to hospital, parents were taught about breastfeeding (such as how is breast milk extracted, stored and transported to the hospital) by nurses. NICU nurses teach parents about breastfeeding every Monday and Thursday afternoon. These practices were designed to encourage mothers to pump breast milk regularly in order to maintain an adequate supply, and for parents to have the correct knowledge of the collection, storage and transport of breast milk. Once received the breast milk, the nurse checked the infant’s information against the label on the storage bottle and used 1 ml syringe to draw up 0.3 ml volume (According to the summary of the preliminary trial that 0.3 ml milk can just soak a medical sterile swab). The syringes stored at 2–6 °C in a specified milk refrigerator, they were available for 24 h. Breast milk used for intestinal feeding was refrigerated or frozen depending on the feeding situation of preterm infants in accordance with our NICU feeding protocol.

### Study design

This randomized controlled clinical trial was conducted from August 2020 to January 2021 in the Neonatal Intensive Care Unit (NICU) of the Shenzhen Maternity&Child Healthcare Hospital, China. All infants (n = 130) were randomized into a experimental group or a control group from 1:1 ratio random number table generated by computer. Of the 130 preterm infants, 19 dropped out of the study due to death or lack of breast milk. The oropharyngeal administration of mother’s milk was started immediately after obtaining breast milk and was given every 3 h for the 14 days. This procedure performed 5 min prior to each gavage feeding. The buccal swab served to evenly distribute the milk over the cheeks, gums, tongue surface and sublingual. Swabs were applied to the oral cavity quickly (< 5 s per side) and gently. The session was discontinued and recorded if any of the following issues developed: bradycardia (HR < 100/min) or tachycardia (HR > 200/min), respiratory rate (RR > 80/min), apnea and pulse oxygen saturation (SpO_2_ < 88%). After the end of the intervention, the infants were continued to be observed for more than 30 s. The control group accepted oropharyngeal administration of 0.9% normal saline every shift. The physician decides the feeding status of the infants. Both groups received breast milk for enteral nutrition with orogastric tube. Formula feeding only when breast milk was insufficient. Total parenteral nutrition was continued until infants were receiving an enteral feeding volume equal to 100 ml/kg/l.

### Demographic and outcome data

Maternal demographics, containing age, prenatal diseases and medication status. Infant demographic information was collected and compared. The first part of the record included gender, gestational age at birth, birth weight, number of babies, Apgar score, delivery mode, etc. The second part of the data document registered the type and dosage of tube-fed milk for premature infants within 14 days, as well as the time and frequency of oropharyngeal administration of mother’s milk.

### Specimen collection and assays

Saliva was collected at the first 2 h, 7th and 14th days of life. The saliva samples on the 7th and 14th days after birth were collected 2 h after tube-feeding in the morning. The samples were centrifuged at 5000 rpm for 20 min and the supernatant were stored in freeze (− 30 °C) until analysis. Before measurement, collection tubes were removed from the freezer and thawed at room temperature for 10–15 min. The concentration of sIgA in the samples were then measured by quantitative enzyme-linked immunosorbent assay (ELISA) (Abnova, Taiwan, China) following the protocol provided by the manufacturer. After the samples were stained, the absorbance (OD value) was measured at 450 nm, then the concentration of sIgA was calculated. The instrument involved in the experiment was the enzyme-label measuring instrument (Muitiskan FC, Shanghai, China).

### Statistics

The SPSS version 26.0 software was used for data analysis. Chi-square test and t test of independent samples were used for clinical data, Mann–Whitney U was used to compare concentration of sIgA between the study and control groups. Spearman rank correlation analysis was examined only for various factors that may affect the concentration of sIgA in the study groups, and the quantile regression analysis was performed. The p value of less than 0.05 was considered statistically significant.

### Institutional Review Board Statement

The study was conducted according to the guidelines of the Declaration of Helsinki, and approved by the Ethics Committee of Shenzhen Maternity & Child Healthcare Hospital (SFYLS[2020]038).

### Informed consent

Informed consent was obtained from all subjects involved in the study.

## Data Availability

Data can be accessible upon request to corresponding author (yangczgd@smu.edu.cn).
